# Emergence of a clinical *Klebsiella pneumoniae* harboring an *acrAB-tolC* in chromosome and carrying the two repetitive tandem core structures for *bla*_KPC-2_ and *bla*_CTX-M-65_ in a plasmid

**DOI:** 10.3389/fcimb.2024.1410921

**Published:** 2024-07-02

**Authors:** Long He, Wenji Wang, Liman Ma, Dongguo Wang, Shanshan Long

**Affiliations:** ^1^ Department of Clinical Laboratory Medicine, Wenling First People’s Hospital, Taizhou, Zhejiang, China; ^2^ School of Life Sciences, Taizhou University, Taizhou, Zhejiang, China; ^3^ School of Medicine, Taizhou University, Taizhou, Zhejiang, China; ^4^ Department of Central Laboratory, Taizhou Municipal Hospital affiliated with Taizhou University, Taizhou, Zhejiang, China; ^5^ Department of Laboratory Medicine, Sichuan Provincial People’s Hospital, University of Electronic Science and Technology, Chengdu, Sichuan, China

**Keywords:** *K. pneumoniae*, SC35 chromosome, SC35 plasmid p1, *acrAB-tolC*, the two repetitive tandem core structures for *bla*_KPC-2_ and *bla*_CTX-M-65_

## Abstract

**Objective:**

The emergence of clinical *Klebsiella pneumoniae* strains harboring *acrAB-tolC* genes in the chromosome, along with the presence of two repetitive tandem core structures for *bla*_KPC-2_ and *bla*_CTX-M-65_ genes on a plasmid, has presented a significant clinical challenge.

**Methods:**

In order to study the detailed genetic features of *K. pneumoniae* strain SC35, both the bacterial chromosome and plasmids were sequenced using Illumina and nanopore platforms. Furthermore, bioinformatics methods were employed to analyze the mobile genetic elements associated with antibiotic resistance genes.

**Results:**

*K. pneumoniae* strain SC35 was found to possess a class A beta-lactamase and demonstrated resistance to all tested antibiotics. This resistance was attributed to the presence of efflux pump genes, specifically *acrAB-tolC*, on the SC35 chromosome. Additionally, the SC35 plasmid p1 carried the two repetitive tandem core structures for *bla*_KPC-2_ and *bla*_CTX-M-65_, as well as *bla*_TEM-1_ with *rmtB*, which shared overlapping structures with mobile genetic elements as In413, Tn*3*, and Tn*As3*. Through plasmid transfer assays, it was determined that the SC35 plasmid p1 could be successfully transferred with an average conjugation frequency of 6.85 × 10^-4^.

**Conclusion:**

The structure of the SC35 plasmid p1 appears to have evolved in correlation with other plasmids such as pKPC2_130119, pDD01754–2, and F4_plasmid pA. The infectious strain SC35 exhibits no susceptibility to tested antibioticst, thus effective measures should be taken to prevent the spread and epidemic of this strain.

## Introduction

The increasing prevalence and global spread of multidrug-resistant bacteria, including in regions such as South/North America, Europe, Africa, the Middle East, South-East Asia, and Oceania, posed a significant threat to human health worldwide (Hamidian and Nigro, 2019). Among these bacteria, carbapenem-resistant *Klebsiella pneumoniae* had emerged as a major cause of infections globally (World Health Organization (WHO), 2014). Plasmids played a crucial role in the dissemination of drug-resistant genes, such as *bla*_KPC-2_ (Zhai et al., 2021). Various types of plasmids had been found to carry *bla*_KPC_, with the IncFIIk plasmid being the most frequently detected and widely reported (Villa et al., 2010; Pitout et al., 2015; Li et al., 2021). The production of carbapenemase enzymes, including KPC, NDM, and OXA-48, was one of the major resistance mechanisms in *K. pneumoniae* (Pitout et al., 2015).

The presence of CTX-M type extended-spectrum beta-lactamases (ESBLs) was another significant factor contributing to bacterial resistance to cephalosporins. These ESBLs had been widely detected in European countries, as well as in Asia and South America (Cantón and Coque, 2006). Mobile genetic factors played an important role in the emergence of multidrug-resistant bacteria (Yu et al., 2024). There were notable variations in the distribution of CTX-M types across different countries and continents (Yu et al., 2024). In Asia, CTX-M-14 and CTX-M-55 were the predominant genotypes, while CTX-M-1 was more prevalent in Europe (Yu et al., 2024), and higher frequency of IncI-I(α) plasmids were found in Eurasian and Asian transmission clusters (Yu et al., 2024). The transfer of CTX-M-14 was mainly mediated by IS*Ecpl*, while IS*26* played a significant role in the transfer of CTX-M-65. Additionally, *bla*_TEM_ and *bla*_CTX-M_ were frequently found together within the same transposable unit (Yu et al., 2024). Studies had reported concurrent involvement of *bla*_KPC-2_, *bla*_CTX-M-65_, and *bla*_SHV-12_ on the same plasmid (Xiang et al., 2016; Liu et al., 2018), indicating a close relationship and a highly similar genetic backbone among these resistance genes (Liu et al., 2018). Sequencing results had demonstrated that IS*26* mediated the duplication of the IS*26*-*fosA3*-*bla*_CTX-M-65_ plasmid insertion element in certain strains (Wang et al., 2023).

Bacterial resistance to antimicrobial drugs could occur through four main mechanisms: (1) Inactivation of the drugs through degradation or chemical modification, (2) Mutations in the targets of the drugs, resulting in reduced binding or efficacy, (3) Changing in membrane permeability, and (4) Active efflux of drugs, reducing intracellular concentrations (Reygaert, 2018). Mechanisms (1) and (2) generally conferred resistance to specific or similar antibiotics, such as KPC or CTX-M-65, which could be present in the same strain, leading to resistance to multiple drugs within their respective antibiotic families (Yamasaki et al., 2023). On the other hand, mechanisms (3) and (4) could increase resistance to multiple structurally unrelated antibiotics (Reygaert, 2018). For example, a single multidrug efflux pump, such as *tmexCD1-toprJ1* (Qu et al., 2023) or *acrAB-tolC*, had the ability to transport multiple structurally unrelated substrates, and its high expression could result in bacterial resistance to various antibiotic families (Yamasaki et al., 2023). Therefore, the presence or acquisition of resistance genes, along with cellular drug efflux pumps or channels, significantly enhanced bacterial multidrug resistance, making clinical treatment more challenging and complex, and posing a greater risk to infected patients.

This study aims to characterize the multidrug resistance genes present on the chromosome harboring *acrAB-tolC* and a plasmid carrying two repetitive tandem core structures for *bla*_KPC-2_ and *bla*_CTX-M-65_. The research will focus on annotating the structure of resistance genes on the plasmid, comparing the structural relationships between similar plasmids, and exploring possible evolutionary processes. The study will provide detailed data that can be used for the treatment of highly multidrug-resistant strains with similar resistance mechanisms.

## Materials and methods

### Bacterial strains and sequencing of the 16S rRNA gene

A 57-year-old man was admitted to the intensive care unit with a closed craniocerebral injury resulting in intracranial and pulmonary infection, but empiric antimicrobial treatment with tigecycline plus polymyxin was ineffective. Subsequently, *K. pneumoniae* SC35, a carbapenem- insensitive strain, was isolated from the patient’s sputum at Taizhou Municipal Hospital affiliated with Taizhou University in 2021, and for this reason, this selected strain was further investigated in depth in this paper.

For species verification, the almost complete 16S rRNA gene was amplified using PCR. The forward primer used was 5’-AGAGTTTGATYMTGGGGCTCAG’, and the reverse primer used was 5’-TACCTTGTTACGACTT-3’ (where Y represents T or C, and M represents A or C). The length of the amplicon obtained was approximately 1,500 bp (Frank et al., 2008). The PCR amplification was performed using a mixture of Fermentas Taq and Pfu enzymes (in a 3:1 ratio) from ThermoFisher Scientific. The reaction mixture contained 30 ml of the enzyme mixture, and the PCR conditions included an initial denaturation step at 94°C for 3 min, followed by denaturation at 94°C for 40 s, annealing at 50°C for 40 s, extension at 72°C for 1 min, and a final extension step at 72°C for 5 min. A total of 30 cycles were performed. To characterize the PCR products, bidirectional sequencing was carried out. This sequencing method allowed for sequencing in both the forward and reverse directions, providing more accurate and reliable results.

### Conjugate transfer and plasmid transfer experiments

#### Conjugation experiments

To perform conjugation, the EC600 receptor strain and SC35 donor strain were mixed together in lysozyme broth (LB), which served as the growth medium. Logarithmic phase cells of both the donor and recipient strains (0.5 mL each) were taken and cultured in 4 mL of fresh LB at 35°C for 18–24 hours without shaking. This incubation period allowed for the transfer of genetic material from the donor to the recipient strain. After incubation, Tryptic Soy Agar (TSA) plate containing selective antibiotics were used to screen for transconjugants. Specifically, the TSA plates were supplemented with 10 μg/L rifampicin and 0.02 μg/L imipenem. These antibiotic concentrations were effective in inhibiting the growth of the donor and recipient strains but allowed the growth of transconjugants that had acquired the resistance genes from the donor strain through conjugation. The transconjugants, which were the result of successful conjugation and acquisition of the resistance genes, could be identified and isolated on the TSA plates supplemented with the selective antibiotics.

#### Plasmid electroporation tests

For plasmid electroporation, *E. coli* DH5α cells were used as the recipient cells for transformation experiments. The conjugation frequency was calculated by determining the number of transduced conjugates per initial donor cell. To prepare the electroactive cells, the bacteria were grown to an optical density (OD600) of 0.5–0.6. The cells were then precipitated by centrifugation at 4°C and washed twice with 1 volume of milliQ water, followed by centrifugation at 6,000 rpm at 4°C. Finally, the cells were centrifuged with 1/50 volume of 10% glycerol. This washing process helped in removing any residual media and preparing the cells for electroporation. The prepared electroactive cells were aliquoted and stored at -70°C for backup. For electroporation, aliquots of the electroactive cells were mixed with less than 10 ng of DNA in a 0.2 cm cuvette. The electroporation was performed using a MicroPulser apparatus (Bio-Rad, California, USA) at specific settings, such as 2.5 kV, 25 mOhm, and 200 Ω. This electrical pulse facilitates the uptake of the plasmid DNA by the recipient cells. After electroporation, the cells were immediately transferred into 1 mL of LB medium and incubated at 37°C with shaking. To ensure the expression of the antibiotic resistance encoded by the plasmids, the cells were incubated in a culture medium containing antibiotics. In the case of strain SC35, the plasmids were properly screened using TSA plates supplemented with 10 μg/L rifampicin and 0.02 μg/L imipenem, which allowed for the selection and growth of cells carrying the plasmids.

### Antimicrobial susceptibility testing

In the bacterial resistance assay, the BioMerieux VITEK2 system was used to determine the Minimum Inhibitory Concentration (MIC) values of various antibiotics (**Table 1**). Additionally, the disk diffusion test was performed to measure the diameter (mm) of the zones of inhibition, indicating bacterial resistance. The results obtained were confirmed using the 2022 Clinical and Laboratory Standards Institute (CLSI) guidelines (Clinical and Laboratory Standards Institute (CLSI), 2022). A total of 33 antibiotics, including antibiotics combined with enzyme inhibitors, were tested. The MIC values obtained from the VITEK2 system and the zones of inhibition from the disk diffusion test were recorded in **Table 1**. To ensure the reliability of the assay, *E. coli* ATCC 25922 was selected as the quality control strain. The MIC values and zone diameter measurements were accurately determined bacterial resistance, and the results obtained from the assay were compared to the breakpoints and interpretive criteria.

**Table 1:** MICs and genetic profiles of *K. pneumoniae* SC35 (ST11).

Antimicrobial agents	MIC (mg/L)	Mechanism of resistance/ location of resistance gene
Aminoglycoside		
Amikacin	≥64.0 (R)	*aadA2*/*acrD*/*kpnEFGH*/*baeR* (**SC35 chromosome**), *aadA5*/*aph(6)-Id*/*aph(3')-Ib*/*rmtB* (**SC35 plasmid p1**)
Gentamycin	≥16.0 (R)	*aadA2*/*acrD*/*kpnEFGH*/*baeR* (**SC35 chromosome**), *aadA5*/*aph(6)-Id*/*aph(3')-Ib*/*rmtB* (**SC35 plasmid p1**)
Tobramycin	≥16.0 (R)	*aadA2*/*acrD*/*kpnEFGH*/*baeR* (**SC35 chromosome**), *aadA5*/*aph(6)-Id*/*aph(3')-Ib*/*rmtB* (**SC35 plasmid p1**)
β-lactams		
Ertapenem	≥8.0 (R)	*ampH*/*bla*_SHV-11_/*oprBDM*/*ompA*/*acrAB-tolC-marR*/*acrAB*/*kpnGH* (**SC35 chromosome**), *bla*_CTX-M-65_/*bla*_KPC-2_/*bla*_TEM-1_ (**SC35 plasmid p1**)
Imipenem	≥16.0 (R)	*ampH*/*bla*_SHV-11_/*oprBDM*/*ompA*/*acrAB-tolC-marA*/*acrAB*/*kpnGH* (**SC35 chromosome**), *bla*_CTX-M-65_/*bla*_KPC-2_/*bla*_TEM-1_ (**SC35 plasmid p1**)
Meropenem	≥16.0 (R)	*ampH*/*bla*_SHV-11_/*CRP*/*oprBDM*/*ompA*/*acrAB-tolC-marR*/*acrAB*/*kpnGH*/*CBP-1* (**SC35 chromosome**), *bla*_CTX-M-65_/*bla*_KPC-2_/*bla*_TEM-1_ (**SC35 plasmid p1**)
Doripenem	≥8.0 (R)	*ampH*/*bla*_SHV-11_/*oprBDM*/*ompA*/*acrAB-tolC-marR*/*acrAB*/*kpnGH* (**SC35 chromosome**), *bla*_CTX-M-65_/*bla*_KPC-2_/*bla*_TEM-1_ (**SC35 plasmid p1**)
Piperacillin	≥128.0 (R)	*ampH*/*PBP3*/*bla*_SHV-11_/*oprBDM*/*ompA*/*acrAB-tolC-marR*/*acrAB*/*kpnEFGH* (**SC35 chromosome**), *bla*_CTX-M-65_/*bla*_KPC-2_/*bla*_TEM-1_ (**SC35 plasmid p1**)
Ticarcillin	≥128.0 (R)	*ampH*/*PBP3*/*bla*_SHV-11_/*oprBDM*/*ompA*/*acrAB-tolC-marR*/*kpnEFGH* (**SC35 chromosome**), *bla*_CTX-M-65_/*bla*_KPC-2_/*bla*_TEM-1_ (**SC35 plasmid p1**)
Aztreonam	≥64.0 (R)	*ampH*/*PBP3*/*bla*_SHV-11_/*oprBDM*/*ompA*/*ompA*/*acrAB-tolC-marR*/*acrAB*/*kpnEFGH* (**SC35 chromosome**), *bla*_CTX-M-65_/*bla*_KPC-2_/*bla*_TEM-1_ (**SC35 plasmid p1**)
Cefazolin	≥64.0 (R)	*ampH*/*PBP3*/*bla*_SHV-11_/*oprBDM*/*ompA*/*acrAB-tolC-marR*/*acrAB*/*kpnEFGH* (**SC35 chromosome**), *bla*_CTX-M-65_/*bla*_KPC-2_/*bla*_TEM-1_ (**SC35 plasmid p1**)
Cephalothin	≥64.0 (R)	*ampH*/*PBP3*/*bla*_SHV-11_/*oprBDM*/*ompA*/*acrAB-tolC-marR*/*acrAB*/*kpnEFGH* (**SC35 chromosome**), *bla*_CTX-M-65_/*bla*_KPC-2_/*bla*_TEM-1_ (**SC35 plasmid p1**)
Cefuroxime	≥64.0 (R)	*ampH*/*PBP3*/*bla*_SHV-11_/*oprBDM*/*ompA*/*acrAB-tolC-marR*/*acrAB*/*kpnEFGH* (**SC35 chromosome**), *bla*_CTX-M-65_/*bla*_KPC-2_/*bla*_TEM-1_ (**SC35 plasmid p1**)
Ceftizoxime	≥64.0 (R)	*ampH*/*PBP3*/*bla*_SHV-11_/*oprBDM*/*ompA*/*acrAB-tolC-marR*/*acrAB*/*kpnEFGH* (**SC35 chromosome**), *bla*_CTX-M-65_/*bla*_KPC-2_/*bla*_TEM-1_ (**SC35 plasmid p1**)
Ceftazidime	≥64.0 (R)	*ampH*/*PBP3*/*bla*_SHV-11_/*oprBDM*/*ompA*/*acrAB-tolC-marR*/*acrAB*/*kpnEFGH* (**SC35 chromosome**), *bla*_CTX-M-65_/*bla*_KPC-2_/*bla*_TEM-1_ (**SC35 plasmid p1**)
Ceftriaxone	≥64.0 (R)	*ampH*/*PBP3*/*bla*_SHV-11_/*oprBDM*/*ompA*/*acrAB-tolC-marR*/*acrAB*/*kpnEFGH* (**SC35 chromosome**), *bla*_CTX-M-65_/*bla*_KPC-2_/*bla*_TEM-1_ (**SC35 plasmid p1**)
Cefotaxime	≥64.0 (R)	*ampH*/*PBP3*/*bla*_SHV-11_/*oprBDM*/*ompA*/*acrAB-tolC-marR*/*acrAB*/*kpnEFGH* (**SC35 chromosome**), *bla*_CTX-M-65_/*bla*_KPC-2_/*bla*_TEM-1_ (**SC35 plasmid p1**)
Cefepime	≥64.0 (R)	*ampH*/*PBP3*/*bla*_SHV-11_/*oprBDM*/*ompA*/*acrAB-tolC-marR*/*acrAB*/*kpnEFGH* (**SC35 chromosome**), *bla*_CTX-M-65_/*bla*_KPC-2_/*bla*_TEM-1_ (**SC35 plasmid p1**)
Cefpodoxime	≥8.0 (R)	*ampH*/*PBP3*/*bla*_SHV-11_/*oprBDM*/*ompA*/*acrAB-tolC-marR*/*acrAB*/*kpnEFGH* (**SC35 chromosome**), *bla*_CTX-M-65_/*bla*_KPC-2_/*bla*_TEM-1_ (**SC35 plasmid p1**)
Amoxicillin/ clavulanic acid	≥32.0 (R)	*ampH*/*PBP3*/*bla*_SHV-11_/*oprBDM*/*ompA*/*acrAB-tolC-marR*/*acrAB*/*kpnEFGH* (**SC35 chromosome**), *bla*_CTX-M-65_/*bla*_KPC-2_/*bla*_TEM-1_ (**SC35 plasmid p1**)
Piperacillin/ Tazobactam	≥128.0 (R)	*ampH*/*PBP3*/*bla*_SHV-11_/*oprBDM*/*ompA*/*acrAB-tolC-marR*/*acrAB*/*kpnEFGH* (**SC35 chromosome**), *bla*_CTX-M-65_/*bla*_KPC-2_/*bla*_TEM-1_ (**SC35 plasmid p1**)
Cefoperazone/ Avibactam	≥16.0 (R)	*ampH*/*PBP3*/*bla*_SHV-11_/*oprBDM*/*ompA*/*acrAB-tolC-marR*/*acrAB*/*kpnEFGH* (**SC35 chromosome**), *bla*_CTX-M-65_/*bla*_KPC-2_/*bla*_TEM-1_ (**SC35 plasmid p1**)
*Cefoperazone/ Sulbactam (75/30 μg)	6 mm (R)	*ampH*/*PBP3*/*bla*_SHV-11_/*oprBDM*/*ompA*/*acrAB-tolC-marR*/*acrAB*/*kpnEFGH* (**SC35 chromosome**), *bla*_CTX-M-65_/*bla*_KPC-2_/*bla*_TEM-1_ (**SC35 plasmid p1**)
Cephamycin		
Cefotetan	≥64.0 (R)	*ampH*/*PBP3*/*bla*_SHV-11_/*oprBDM*/*ompA*/*acrAB-tolC-marR*/*acrAB*/*kpnEFGH* (**SC35 chromosome**), *bla*_CTX-M-65_/*bla*_KPC-2_/*bla*_TEM-1_ (**SC35 plasmid p1**)
Fluoroqinolones		
Levofloxacin	≥8.0 (R)	*acrAB-tolC-marR*/*acrAB*/*parC*/*CRP*/*rmsA*/*knpGH*/*emrR* (**SC35 chromosome**)
Ciprofloxacin	≥4.0 (R)	*acrAB-tolC-marR*/*acrAB*/*parC*/*CRP*/*rmsA*/*knpGH*/*emrR* (**SC35 chromosome**)
Moxifloxacin	≥8.0 (R)	*acrAB-tolC-marR*/*acrAB*/*parC*/*CRP*/*rmsA*/*knpGH*/*emrR* (**SC35 chromosome**)
Tetracycline		
Tetracycline	≥16.0 (R)	*acrAB-tolC-marR*/*acrAB*/*knpEF*/*mdfA* (**SC35 chromosome**), *tet(A)* (**Plasmid SC35-2**)
Polymyxin		
*Polymyxin B (300 μg)	6 mm (R)	*eptB*/*pmrF*/*arnT*/*lptD* (**SC35 chromosome**)
Fosfomycin		
*Fosfomycin (50 μg)	6 mm (R)	*uhpT*/*fosA6* (**SC35 chromosome**)
Glycylcycline		
Tigecycline	≥8 (R)	*acrAB-tolC-marR*/*acrAB* (**SC35 chromosome**)
Macrolide		
*Erythromycin (15 μg)	6 mm (R)	*CRP*/*kpnEFGH* (**SC35 chromosome**), *mphA* (**SC35 plasmid p1**)
Sulfonamide		
Trimethoprim- sulfamethoxazole	≥320 (R)	*sul1* (**SC35 chromosome**), *sul1*/*sul2* (**SC35 plasmid p1**)
Disinfecting agent and antiseptic		
–	–	*qacEdelt1*/*gyrA*/ *acrAB-tolC-marA*/*acrAB*/*mdfA* (**SC35 chromosome**), *qacEdelt1* (**SC35 plasmid p1**)

*Disc diffusion method; R, drug-resistance.

### Confirmation of beta-lactamases

#### Confirmation test for ESBLs

According to the CLSI Guidelines 2022, the presence of extended-spectrum beta-lactamases (ESBLs) was determined based on the difference in the diameter of the inhibition zone for specific groups of drugs. Specifically, positive ESBL production was confirmed if the difference in the circle of inhibition diameter was equal to or greater than 5 mm for any of the following drug groups: ceftazidime (CAZ, 30 µg) and ceftazidime/clavulanic acid (30/30 µg), ceftazidime (CTX, 30 µg) and ceftizoxime/clavulanic acid (30/30 µg). This criterion was used to identify bacteria that produced ESBLs, which were enzymes capable of hydrolyzing and inactivating a broad range of beta-lactam antibiotics, including cephalosporins and monobactams.

#### Determination for classes B and D beta-lactamases

According to the CLSI Guidelines 2022 (Clinical and Laboratory Standards Institute (CLSI), 2022), the modified carbapenem inactivation method (mCIM) and the modified carbapenem inactivation method + EDTA (eCIM) were used to determine the presence of metal-β-lactamases, specifically class B carbapenemases. In the mCIM, a carbapenem antibiotic was combined with a EDTA to inhibit the activity of metallo-β-lactamases (MBLs). If there was no MBL present, the carbapenem antibiotic was able to inhibit the bacterial growth, resulting in a zone of inhibition. If the addition of EDTA increased the zone of inhibition by 5 mm or more compared to the mCIM, it was considered positive for metal-β-lactamases (class B carbapenemases).

On the other hand, the identification of the class D carbapenemase phenotype, known as class D β- lactamases, currently lacked a definitive method. However, it could be inferred by a measure of exclusion. If the strain did not exhibit inhibition by class A or B inhibitors, it was inferred to possess a class D carbapenemase.

### Sequencing and sequence assembly

The genome of strain SC35 was sequenced using two different sequencing platforms, PacBio Sequel II and Illumina NovaSeq 6000. The DNA libraries used had an average size of 15 kb for PacBio Sequel II and 400 bp for Illumina NovaSeq 6000. Raw PacBio Sequel II and Illumina reads were trimmed using Canu v2.2 (https://github.com/marbl/canu) and Trimmomatic v0.39 (https://github.com/usadellab/Trimmomatic), respectively. After trimming, 13.7 million clean Illumina reads were obtained with an average length of 148 bp and 100% of reads >=Q20. The paired-end short Illumina reads were then assembled using either Unicycler v0.4.5 (https://github.com/rrwick/Unicycler) or SPAdes v3.15.3 (https://github.com/ablab/spades) to improve the accuracy of data processing. Finally, the post-assembly sequence corrections were performed on the Illumina reads using Pilon v1.24 (https://github.com/broadinstitute/pilon). This comprehensive approach resulted in the generation of accurate DNA sequences for strain SC35.

### Sequence annotation and comparison in detail

The prediction of open reading frames (orfs) and pseudogenes was performed using *RAST 2.0* (Brettin et al., 2015), *BLASTP/BLASTN* (Boratyn et al., 2013), *UniProtKB/Swiss-Prot* (Boutet et al., 2016), and *RefSeq databases* (O’Leary et al., 2016). Fine annotation of drug-resistance genes, mobile elements, and other features was undertaken through online databases such as *CARD 2023* (Alcock et al., 2023), *ResFinder 4.0* (Bortolaia et al., 2020), IS*finder* (Siguier et al., 2006), *INTEGRALL* (Moura et al., 2009), and the Tn *Number Registry* (Tansirichaiya et al., 2019). Cloned MLSTs were identified using *MLST 2.0* (https://cge.food.dtu.dk/services/MLST/) and *BacWGSTdb 2.0* (Feng et al., 2021). The SC35 plasmid p1 and its related plasmids were compared for multiple and pairwise sequence comparisons using *MUSCLE 3.8.31* (Edgar, 2004) and *BLASTN*, respectively. Circular plots of the chromosome and plasmids were produced by *CGView* (Stothard et al., 2019). All plasmids comparison figures were created using the R package *genoPlotR v0.8.11* software (http://genoplotr.r-forge.r-project.org/) and edited using *Inkscape v0.48.1* (https://inkscape.org/en).

### Nucleotide sequence accession numbers

The sequences of the SC35 chromosome, SC35 plasmid p1, SC35 plasmid p2, SC35 plasmid p3, and SC35 plasmid p4 were deposited on the GenBank database with the accession numbers CP147174.1, CP147175.1, CP147176.1, CP147177.1, and CP147178.1, respectively. The characterization of the related plasmids compared with the SC35 plasmid p1 and F4_plasmid pB could be found in **Table 2**.

**Table 2:** Profiles of the *K. pneumoniae* plasmids studied in the paper.

No.	Plasmid	Isolate source	Collection date	Type	Size (kbp)	GC%	status	GenBank accession no.
1	SC35 plasmid p1	Patient’s sputum (China: Taizhou)	2021-04	IncFII	143.791	53.12	Complete	CP147175.1
2	pKPC2_130119	Patient’s racheal Secretion (China: Chengdu)	2019-03	IncFII	153.000	53.11	Complete	CP127230.1
3	pKPC2_115069	Patient (China: Chengdu)	2018	IncFII	154.986	53.05	Complete	CP033404.1
4	pKPC2_020003	Patient (China: Chengdu)	2016-09	IncFII	154.957	53.07	Complete	CP031720.1
5	pKPC2_095649	Patient’s secreta (China: Chengdu)	2015-11	IncFII	156.099	53.00	Complete	CP026584.1
6	p21072329_1	Patient’s sputum (China: Hefei)	2021-07	–	173.108	53.71	Complete	CP095235.1
7	pXHKP6-1	Patient’s venipuncture tube (China: Shanghai)	2010-09	IncFII	180.300	53.90	Complete	CP066888.1
8	pBSI014-KPC2	Patient’s blood (China: Guangzhou)	2016-09	IncFII	170.701	53.57	Complete	MT269822.1
9	pDD01754-2	Patient (China: Shanghai)	2017-12	IncFII	156.099	53.36	Complete	CP087647.1
10	F94_Plasmid pA	Patient’s sputum (China: Taizhou)	2021-01	IncFII	172.743	53.57	Complete	OM144977.1

## Results

### Antimicrobial susceptibility testing, enzymatic characterization and transferable properties

The strain SC35 was identified as *K. pneumoniae* through BLAST analysis of its 16S rRNA and genome sequences, as well as through nucleotide homology analysis. **Table 1** provided the MIC values and circle of inhibition diameters of the strain SC35 in the drug susceptibility testing conducted using BioMerieux VITEK2 and the paper diffusion method. The strain SC35 exhibited resistance to all tested antibiotics (**Table 1**).

Enzyme characterization revealed that the strain SC35 produced class A beta-lactamase. The MLST sequence was characterized as sequence type (ST) 11 using *MLST 2.0* and *BacWGSTdb 2.0*. Through bacterial conjugation transfer and electroporation experiments, the integrated SC35 plasmid p1 was successfully recovered, with an average conjugation frequency of 6.85 × 10^-4^.

### Investigation and characterization of drug-resistance genes on the SC35 chromosome and SC35 plasmids

**Figure 1:**
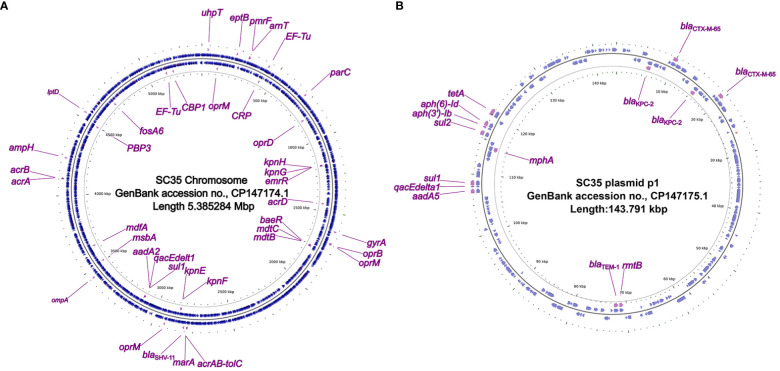
Circos plot of the SC35 chromosome and SC35 plasmid p1 and localization of antibiotic resistance genes. **(A)** The SC35 chromosome of 5.385284 Mbp in length showing antibiotic resistance genes. **(B)** The SC35 plasmid p1 of 143.791 kbp in length showing antibiotic resistance genes. The Circos plot of F4_chromosome was established using *CGview v2.0.3* (https://github.com/paulstothard/cgview). The locations of the antibiotic resistance genes were drawn manually using *Inkscape 0.48.1* (https://inkscape.org/en).

In the strain SC35, a chromosome and four plasmids were identified, including the SC35 plasmid p1, SC35 plasmid p2, SC35 plasmid p3, and SC35 plasmid p4. On the SC35 chromosome, a total of 40 different genes associated with drug resistance were found. These genes included drug-resistant nodule disintegration (RND) antibiotic efflux pumps (e.g., *acrAB-tolC*), major facilitator superfamily (MFS) antibiotic efflux pumps (e.g., *kpnGH*), and ATP-binding cassette (ABC) antibiotic efflux pumps (e.g., *msbA*) (**Figure 1A**). For example, it had been documented that upregulation of the expression of the AcrAB-TolC was the major cause of *marA*-mediated MDR (Li and Nikaido, 2004), whereas AcrAB-TolC overexpression significantly improved the response of *marA*-mediated MDR to all antibiotics (Ruiz and Levy, 2010). The characterization of drug-resistance genes on the SC35 chromosome could be found in **Table 3**.

**Table 3:** Characterization of drug-resistance genes located on SC35 chromosome.

Drug-resistance gene	Start	Stop	Orientation	Identities (%)	Sequence length (%)	Protein model-type	SNPs	Drug Class	Resistance Mechanism	Antimicrobiol resistance Gene Family
*uhpT* mutants	39186	40577	+	95.03	100	variant	E350Q	fosfomycin	antibiotic target alteration	antibiotic-resistant UhpT
*eptB*	220412	222085	+	99.46	97.04	homolog	n/a	peptide antibiotic	antibiotic target alteration	pmr phosphoethanolamine transferase
*oprM*	105873	107237	–	100	100	homolog	n/a	monobactam; carbapenem; cephalosporin; cephamycin; penam; penem	reduced permeability to antibiotic	General Bacterial Porin with reduced permeability to beta-lactams
*pmrF*	298204	299187	+	83.69	101.55	homolog	n/a	peptide antibiotic	antibiotic target alteration	pmr phosphoethanolamine transferase
*arnT*	302068	303723	+	99.64	100	homolog	n/a	peptide antibiotic	antibiotic target alteration	pmr phosphoethanolamine transferase
*CRP*	414796	415428	–	99.05	100	homolog	n/a	macrolide; fluoroquinolone; penam	antibiotic efflux	resistance-nodulation-cell division (RND) antibiotic efflux pump
*EF-Tu* mutants	430475	431659	+	97.97	96.33	variant	R234F	elfamycin antibiotic	antibiotic target alteration	elfamycin resistant EF-Tu
*parC* mutants	714034	716292	+	94.41	100	variant	S80I	fluoroquinolone antibiotic	antibiotic target alteration	fluoroquinolone resistant parC
*oprD*	832888	834156	–	100	100	homolog	n/a	monobactam; carbapenem; cephalosporin; cephamycin; penam; penem	reduced permeability to antibiotic	General Bacterial Porin with reduced permeability to beta-lactams
*rsmA*	1167531	1167716	+	85.25	100	homolog	n/a	fluoroquinolone; diaminopyrimidine antibiotic; phenicol antibiotic	antibiotic efflux	RND antibiotic efflux pump
*KpnH*	1175576	1177114	–	94.02	100	homolog	n/a	macrolide; fluoroquinolone; aminoglycoside; carbapenem; cephalosporin; penam; peptide antibiotic; penem	antibiotic efflux	major facilitator superfamily (MFS) antibiotic efflux pump
*KpnG*	1177130	1178302	–	99.74	100	homolog	n/a	macrolide; fluoroquinolone; aminoglycoside; carbapenem; cephalosporin; penam; peptide antibiotic; penem	antibiotic efflux	MFS antibiotic efflux pump
*emrR*	1178428	1178958	–	92.57	100	homolog	n/a	fluoroquinolone antibiotic	antibiotic efflux	MFS antibiotic efflux pump
*acrD*	1445076	1448189	–	91.03	100	homolog	n/a	aminoglycoside antibiotic	antibiotic efflux	RND antibiotic efflux pump
*gyrA* mutants	1614779	1617412	+	96.08	99.89	variant	D87G:2851, S83I:1545, D87G:1545	disinfecting agents and antiseptics	antibiotic target alteration	triclosan resistant gyrA
*oprB*	1691890	1693254	+	100	100	homolog	n/a	monobactam; carbapenem; cephalosporin; cephamycin; penam; penem	reduced permeability to antibiotic	General Bacterial Porin with reduced permeability to beta-lactams
*oprM*	1697930	1699369	+	100	100	homolog	n/a	monobactam; carbapenem; cephalosporin; cephamycin; penam; penem	reduced permeability to antibiotic	General Bacterial Porin with reduced permeability to beta-lactams
*baeR*	1750099	1750821	–	91.67	100	homolog	n/a	aminoglycoside antibiotic; aminocoumarin antibiotic	antibiotic efflux	RND antibiotic efflux pump
*mdtC*	1753710	1756787	–	91.71	100	homolog	n/a	aminocoumarin antibiotic	antibiotic efflux	RND antibiotic efflux pump
*mdtB*	1756788	1759910	–	90.1	100	homolog	n/a	aminocoumarin antibiotic	antibiotic efflux	RND antibiotic efflux pump
*AcrAB-TolC* with *MarR*	2787053	2787487	+	83.33	100	overexpression	n/a	fluoroquinolone; cephalosporin; glycylcycline; penam; tetracycline; rifamycin; phenicol antibiotic; disinfecting agents and antiseptics	antibiotic target alteration; antibiotic efflux	RND antibiotic efflux pump
*marA*	2787508	2787882	+	92.74	97.64	homolog	n/a	fluoroquinolone; monobactam; carbapenem; cephalosporin; glycylcycline; cephamycin; penam; tetracycline; rifamycin; phenicol antibiotic; penem; disinfecting agents and antiseptics	antibiotic efflux; reduced permeability to antibiotic	RND antibiotic efflux pump; General Bacterial Porin with reduced Permeability to beta-lactams
*bla*_SHV-11_	2807984	2808844	+	100	100	homolog	n/a	carbapenem; cephalosporin; penam	antibiotic inactivation	SHV beta-lactamase
*kpnF*	2838999	2839328	–	100	100	homolog	n/a	macrolide; aminoglycoside; cephalosporin; tetracycline; peptide antibiotic; rifamycin; disinfecting agents and antiseptics	antibiotic efflux	MFS antibiotic efflux pump
*kpnE*	2839315	2839677	–	99.17	100	homolog	n/a	macrolide; aminoglycoside; cephalosporin; tetracycline; peptide antibiotic; rifamycin; disinfecting agents and antiseptics	antibiotic efflux	MFS antibiotic efflux pump
*oprM*	2928140	2929519	+	100	100	homolog	n/a	monobactam; carbapenem; cephalosporin; cephamycin; penam; penem	reduced permeability to antibiotic	General Bacterial Porin with reduced permeability to beta-lactams
*sul1*	3108950	3109525	–	100	68.46	homolog	n/a	sulfonamide antibiotic	antibiotic target replacement	sulfonamide resistant sul
*qacEdelta1*	3109519	3109866	–	100	100	homolog	n/a	disinfecting agents and antiseptics	antibiotic efflux	MFS antibiotic efflux pump
*aadA2*	3110030	3110809	–	100	100	homolog	n/a	aminoglycoside antibiotic	antibiotic inactivation	ANT(3'')
*ompA*	3453664	3454734	+	99.44	95.19	homolog	n/a	monobactam; carbapenem; cephalosporin; cephamycin; penam; penem	reduced permeability to antibiotic	General Bacterial Porin with reduced permeability to beta-lactams
*msbA*	3504105	3505853	–	92.78	100	homolog	n/a	nitroimidazole antibiotic	antibiotic efflux	ATP-binding cassette (ABC) antibiotic efflux pump
*mdfA*	3625773	3627005	–	85.61	100	homolog	n/a	tetracycline antibiotic; disinfecting agents and antiseptics	antibiotic efflux	MFS antibiotic efflux pump
*acrA*	4105062	4106255	+	95.24	99.75	homolog	n/a	fluoroquinolone; cephalosporin; glycylcycline; penam; tetracycline; rifamycin; phenicol antibiotic; disinfecting agents and antiseptics	antibiotic efflux	RND antibiotic efflux pump
*acrB*	4106278	4109424	+	91.42	99.9	homolog	n/a	fluoroquinolone; cephalosporin; glycylcycline; penam; tetracycline; rifamycin; phenicol antibiotic; disinfecting agents and antiseptics	antibiotic efflux	RND antibiotic efflux pump
*ampH*	4235026	4236186	+	85.71	100.26	homolog	n/a	cephalosporin; penam	antibiotic inactivation	ampC-type beta-lactamase
*PBP_3_* mutants	4494209	4495975	–	52.37	96.39	variant	D350N, S357N	cephalosporin; cephamycin; penam	antibiotic target alteration	Penicillin-binding protein mutations conferring resistance to beta-lactam antibiotics
*LptD*	4541675	4544023	+	100	100	homolog	n/a	carbapenem; peptide antibiotic; aminocoumarin antibiotic; rifamycin antibiotic	antibiotic efflux	ABC antibiotic efflux pump
*fosA6*	4701466	4701885	–	97.84	100	homolog	n/a	fosfomycin	antibiotic inactivation	fosfomycin thiol transferase
*EF-Tu* mutants	5127810	5128994	–	97.97	96.33	variant	R234F	elfamycin antibiotic	antibiotic target alteration	elfamycin resistant EF-Tu
*CBP-1*	5178244	5178447	–	100	21.75	homolog	n/a	penam	antibiotic inactivation	CBP beta-lactamase

**Figure 2:**
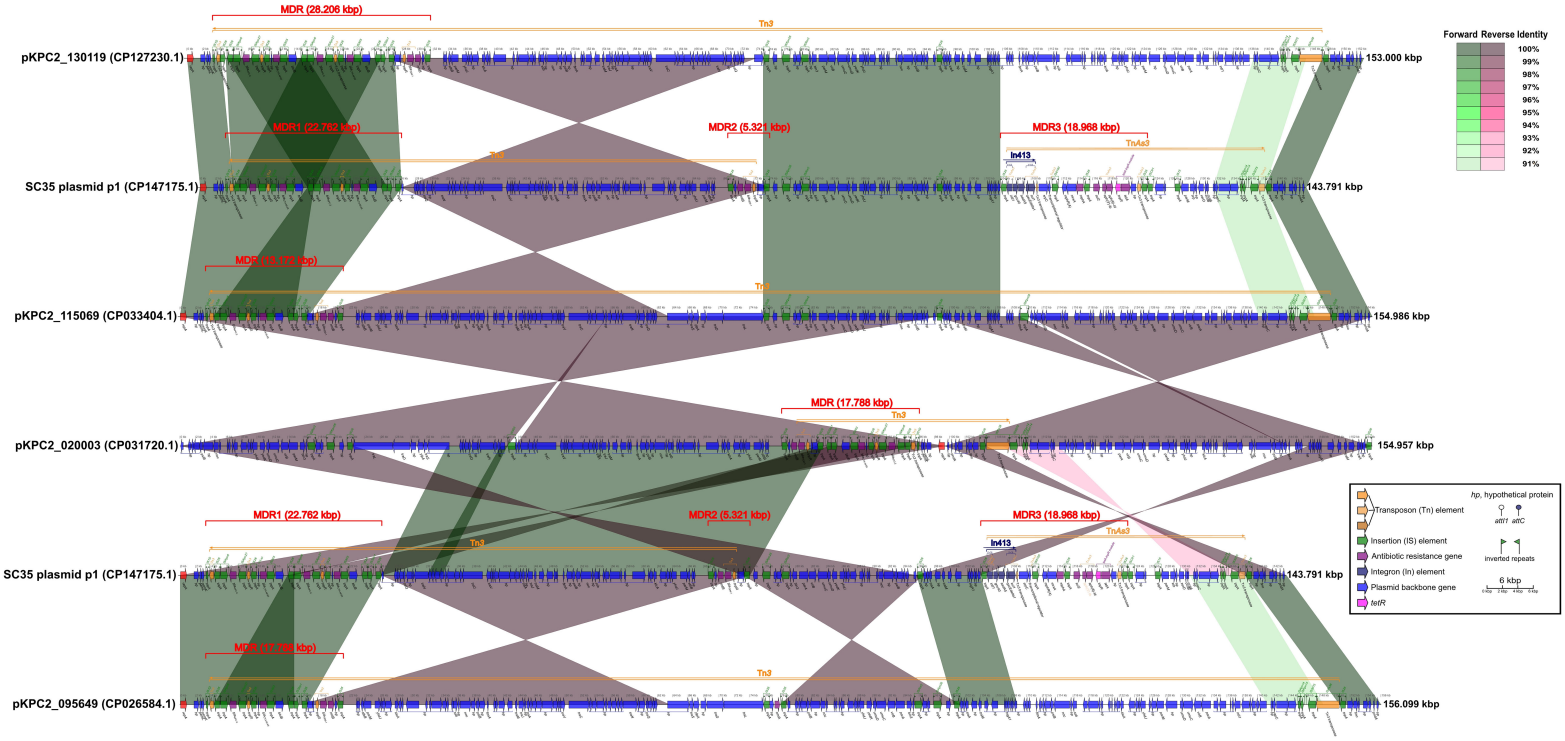
Comparison of structures for SC35 plasmid p1 with closely related plasmids pKPC2_ 130119, pKPC2_115069, pKPC2_020003, and pKPC2_095649. The figure was created by the R package *genoPlotR v0.8.11* software (http://genoplotr.r-forge.r-project.org/).

**Figure 3:**
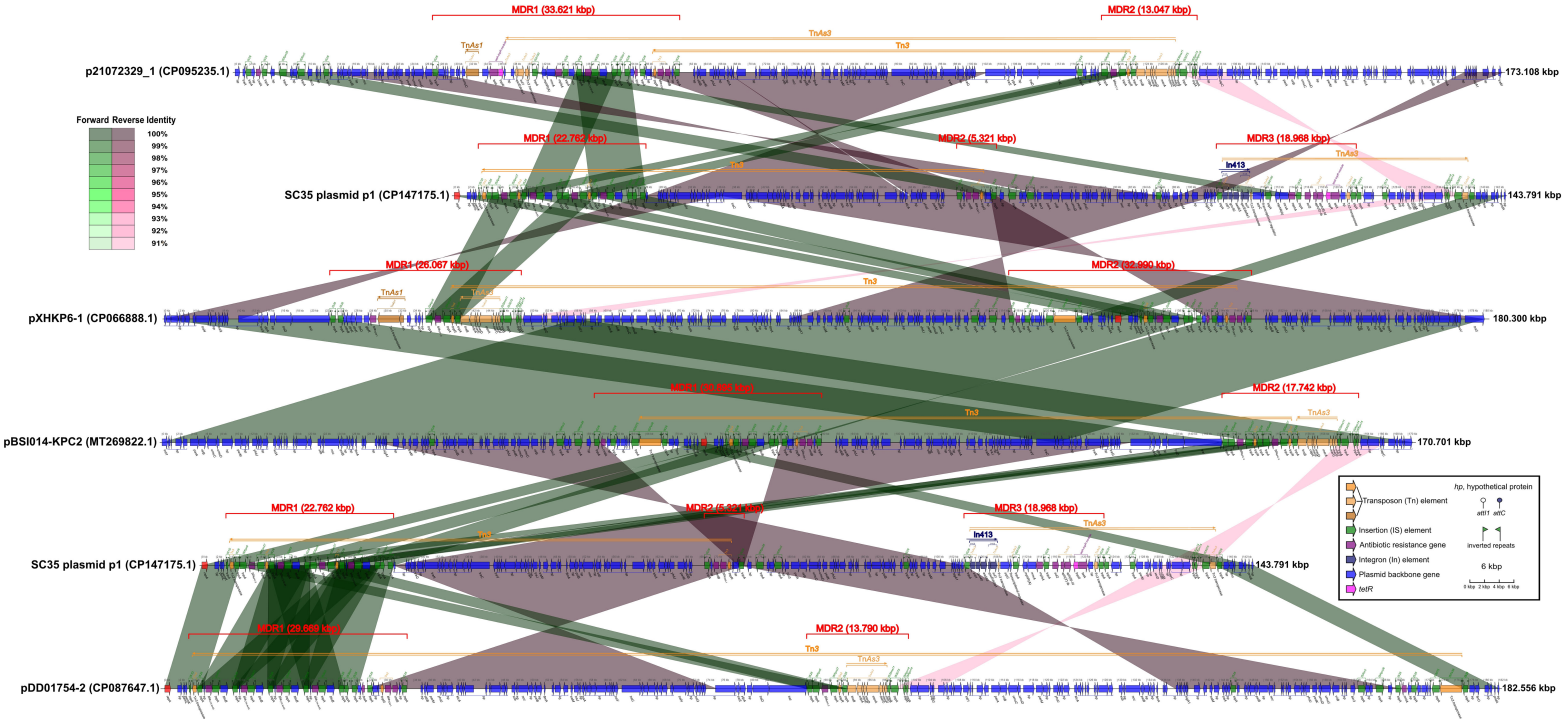
Comparison of structures for SC35 plasmid p1 with other related plasmids p21072329_1, pXHKP6–1, pBSI014-KPC2, and pDD01754–2, China, harboring core structures for *bla*_KPC-2_ and *bla*_CTX-M-65_. The figure was created by the R package *genoPlotR v0.8.11* software (http://genoplotr.r-forge.r-project.org/).

In the SC35 plasmid p1, a total of 14 drug-resistance genes were identified (**Figure 1B**). These genes conferred resistance to β-lactams, aminoglycosides, cephalosporins, tetracyclines, macrolides, sulfonamides, disinfectants, and antiseptics (**Tables 2**, **3**). Further characterization of these resistance genes would be addressed later (**Figures 2**, **3**). However, no resistance genes were found in the SC35 plasmids p2, p3, and p4 (**Supplementary Figure S1**).

### Comparison of structures for the SC35 plasmid p1 with closely related plasmids, pKPC2_ 130119, pKPC2_115069, pKPC2_020003, and pKPC2_095649, Involving the core structures for *bla*_KPC-2_ and *bla*_CTX-M-65_ from Chengdu, China

The SC35 plasmid p1 contained three multidrug-resistant (MDR) regions, namely MDR1, MDR2, and MDR3. The MDR1 region had a sequence length of 22.762 kbp, ranging from 3.383 kbp to 26.144 kbp. It consisted of the two repetitive tandem core structures for *bla*_KPC-2_ and *bla*_CTX-M-65_, which were double repeats of the genetic array of IS*26*-IS*Kpn6*-*bla*_KPC-2_-IS*Kpn27*-*tnpR*-IS*26*-*hp*- *bla*_CTX-M-65_-IS*903*-*btuB-*IS*26* (**Figure 2**). The MDR2 region had a sequence length of 5.321 kbp, ranging from 68.642 kbp to 73.962 kbp, and contained the genetic array IS*26*-*hp*-*rmtB*- *bla*_TEM-1_-*tnpR*-*hp*-*hp*-IS*26*. The MDR3 region had a sequence length of 18.968 kbp, ranging from 104.056 kbp to 123.023 kbp, and included In413 as well as the antibiotic resistance genes *mphA*, *sul2*, *aph(3’)-Ib*, *aph(6)-Id* and *tetA* (**Figure 2**). The structure of SC35 plasmid p1 was characterized by the overlap and intersection of mobile genetic elements as Tn*3*, Tn*As3*, and In413 (**Figure 2**). Comparison of the SC35 plasmid p1 with pKPC2_130119 showed that the MDR1 region of SC35 plasmid p1 shared excellent identities with the MDRs of both pKPC2_130119 and pKPC2_115069 (**Figure 2**). The MDR2 region of SC35 plasmid p1 was inversely identified with the posterior regions of the MDR of pKPC2_130119, ranging from 26.918 kbp to 31.518 kbp (**Figure 2**). The MDR3 region of SC35 plasmid p1 had no identities with the MDRs of pKPC2_130119 and pKPC2_115069 (**Figure 2**). Similarly, the MDR1 region of SC35 plasmid p1 was identical to the MDRs of pKPC2_ 115069 and pKPC2_095649, respectively, only in a genetic array of IS*26*-IS*Kpn*6-*bla*_KPC-2_-IS*Kpn27*- *tnpR*-IS*26*-*hp*-*bla*_CTX-M-65_-IS*903*-*btuB*-IS*26* (**Figure 2**). The MDR2 region of SC35 plasmid p1 was inversely identified with the posterior regions of the MDRs of pKPC2_115069 and pKPC2_095649, ranging from 15.850 kbp to 21.170 kbp (**Figure 2**). The MDR3 region of SC35 plasmid p1 had no identities with the MDRs of pKPC2_115069 and pKPC2_095649 (**Figure 2**). The MDR1 region of SC35 plasmid p1 had inversely extreme identity with the MDR of pKPC2_020003, ranging from 82.857 kbp to 96.028 kbp (**Figure 2**). The MDR2 region of SC35 plasmid p1 was inversely identified with a portion of the MDR of pKPC2_020003, ranging from 78.241 kbp to 83.561 kbp. The MDR3 region of SC35 plasmid p1 also had no identity with the MDR of pKPC2_020003 (**Figure 2**).

### Comparison of structures for the SC35 plasmid p1 with other related plasmids p21072329_1, pXHKP6–1, pBSI014-KPC2, and pDD01754–2, China, harboring the core structures for *bla*_KPC-2_ and *bla*_CTX-M-65_

When SC35 plasmid p1 was compared to p21072329_1, pXHKP6–1, and pBSI014- KPC2, one of the two *bla*_CTX-M-65_ core structures (IS*26*-*hp*-*bla*_CTX-M-65_-IS*903*-*btuB*- IS*26*) in the MDR of SC35 plasmid p1 was identical to a *bla*_CTX-M-65_ core structure in the MDR regions of p21072329_1, pXHKP6–1, and pBSI014-KPC2 (**Figure 3**). One of the two *bla*_KPC-2_ core structures (IS*26*-IS*Kpn6*-*bla*_KPC-2_-IS*Kpn27* -*tnpR*-IS*26*) in the MDR region of SC35 plasmid p1 was almost identical to a *bla*_KPC-2_ core structure in the MDR regions of p21072329_1, pXHKP6–1, and pBSI014-KPC2. However, the arrangement of *bla*_KPC-2_ or *bla*_CTX-M-65_ core structure differed between the SC35 plasmid p1, p21072329_1, pXHKP6–1, and pBSI014-KPC2 (**Figure 3**). Additionally, a genetic array of *hp*-*fosA*-IS*26*-*hp*-*hp*-*tnpR*-*bla*_TEM-1_-*rmtB*-*hp*-IS*26* followed the *bla*_CTX-M-65_ core structure in p21072329_1, pXHKP6–1, and pBSI014-KPC2, but was absent in the SC35 plasmid p1 (**Figure 3**). The MDR3 region of SC35 plasmid p1 also had no identity with the MDR regions of p21072329_1, pXHKP6–1, and pBSI014- KPC2 (**Figure 3**). When the SC35 plasmid p1 was compared to pDD01754–2, one of the two *bla*_CTX-M-65_ core structures (IS*26*-*hp*-*bla*_CTX-M-65_-IS*903*-*btuB*-IS*26*) in the MDR of SC35 plasmid p1 was identical to one of the four repetitive tandem *bla*_CTX-M-65_ core structures in the MDR of pDD01754–2. Either of the two *bla*_CTX-M-65_ core structures (IS*26*-*hp*-*bla*_CTX-M-65_-IS*903*-*btuB*-IS*26*) in the MDR region of SC35 plasmid p1 was identical to either of the four repetitive tandem *bla*_CTX-M-65_ core structures in the MDR region of pDD01754–2 (**Figure 3**). One of the two *bla*_KPC-2_ core structures (IS*Kpn6*-*bla*_KPC-2_-IS*Kpn27*-*tnpR*-IS*26*) in the MDR region of SC35 plasmid p1 was identical to a *bla*_KPC-2_ core structure in the MDR region of pDD01754–2 (**Figure 3**). The MDR3 region of SC35 plasmid p1 also had no identity with the MDR region of pDD01754–2 (**Figure 3**).

### Exploring the structural relationship between SC35 plasmid p1, pDD01754.2 and F4_plasmid pA

**Figure 4:**
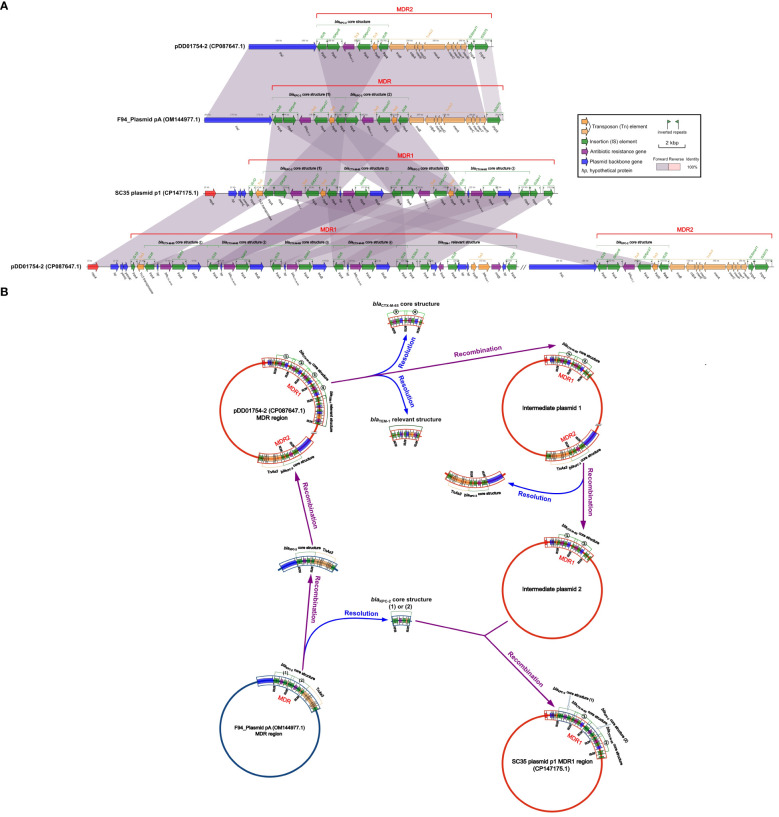
Structural relationship diagram between SC35 plasmid p1, pDD01754.2 and F4_plasmid pA. **(A)** Comparison of structures btween SC35 plasmid p1, pDD01754.2 and F4_plasmid pA. The figure was created by the R package *genoPlotR v0.8.11* software (http://genoplotr.r-forge.r-project.org/). **(B)** Inference plot of the relationship between SC35 plasmid p1, pDD01754.2 and F4_plasmid pA. The figure was created with hand finishing.

By comparing the SC35 plasmid p1 with pDD01754.2 and F4_plasmid pA, it was found that there were good identities between the MDR regions of SC35 plasmid p1, pDD01754.2, and F4_plasmid pA (**Figure 4A**). The sequence structure of F4_plasmid pA from 68.014 kbp to 90.440 kbp was nearly identical to that of pDD01754.2 from 82.573 kbp to 100.253 kbp, except for an additional *bla*_KPC-2_ core structure in F4_plasmid pA (IS*26*-IS*Kpn6*-*bla*_KPC-2_-IS*Kpn27*-*tnpR*-IS*26*) (**Figure 4A**). Either of the two *bla*_KPC-2_ core structures on the SC35 plasmid p1 matched exactly with either of the two tandem *bla*_KPC-2_ core structures on F4_plasmid pA (**Figure 4A**).

Based on the consistency of the structures of SC35 plasmid p1 with pDD01754.2 and F4_plasmid pA (**Figure 4A**), genetic inferences were made as shown in **Figure 4B**. In the evolutionary processes, under suitable conditions, two of the four repetitive tandem *bla*_CTX-M-65_ core structures (③ and ④) and the *bla*_TEM-1_ related structures (*hp*-*fosA*-IS*26*-*hp*-*hp*-*tnpR*-*bla*_TEM-1_-*rmtB*-*hp*-IS*26*) were detached from the MDR1 region of pDD01754.2, forming an intermediate plasmid 1 (**Figure 4B**). Next, the MDR2 region of intermediate plasmid 1, which contained the *bla*_KPC-2_ core structure, was delinked, resulting in the development of an intermediate plasmid 2 that only had two repetitive tandem *bla*_CTX-M-65_ core structures (① and ②) (**Figure 4B**). Subsequently, F4_plasmid pA, which contained the two repetitive tandem *bla*_KPC-2_ core structures, underwent disassociation into one *bla*_KPC-2_ core structure (1) or (2), or two *bla*_KPC-2_ core structures (1) and (2), along with a *bla*_KPC-2_ core structure + Tn*As3* (**Figure 4B**). The resolved *bla*_KPC-2_ core structures (1) and (2) were then recombined with intermediate plasmid 2, which contained the two repetitive tandem *bla*_CTX-M-65_ core structures (① and ②). A *bla*_KPC-2_ core structure (1) was integrated in front of *bla*_CTX-M-65_ ①, and another *bla*_KPC-2_ core structure (2) was inserted in front of *bla*_CTX-M-65_ ②, resulting in the formation of a novel MDR1 region. This novel MDR1 region corresponds to the MDR1 region of SC35 plasmid p1 (**Figure 4B**). Simultaneously, the resolved *bla*_KPC-2_ core structure + Tn*As3* was recombined into pDD01754.2, and the MDR2 region of pDD01754.2 was reorganized (**Figure 4B**).

## Discussion

The *acrAB-tolC* resistance-nodulation-cell division (RND) antibiotic efflux pump, composed of the extracellular cytoplasmic fusion protein AcrA, the drug proton transporter AcrB, and the outer membrane channel protein TolC, was responsible for bacterial resistance to various agents including fluoroquinolones, tetracyclines, cephalosporins, glycylcyclines, penams, rifamycins, phenicol antibiotics, disinfectants, and antiseptics (Li et al., 2015) (**Table 3**). The expression of the *marA* gene, which regulated the transcription of the *acrAB* system, significantly increased the activity of the efflux pump by more than 2.8-fold and induced resistance to a wide range of antibiotics (Shi et al., 2022). In the SC35 strain, the presence of *acrAB-tolC* on the chromosome led to a high level of resistance to various antibiotics including fluoroquinolones, tetracyclines, cephalosporins, cephalomycins, meropenem, and glycylcyclines (tigecycline) (**Table 1**).

In addition to the RND efflux pump, the SC35 chromosome also contained MFS antibiotic efflux pump and ABC antibiotic efflux pump (**Table 3**). These efflux pumps contributed to the resistance phenotype of the SC35 strain. Furthermore, the presence of various non-efflux pump resistance genes on both the SC35 chromosome and SC35 plasmid p1, along with the overlapping and interaction of mobile genetic elements as In413, Tn*3*, and Tn*As3* on the SC35 plasmid p1 (**Figures 1**–**3**), further contributed to the emergence of a highly multidrug-resistant strain SC35 (**Table 1**). The combination of these resistance mechanisms resulted in the almost complete resistance observed in the strain SC35.

In China, ST11 had been the most prevalent carbapenem-resistant strain of *K. pneumoniae*, and the majority of these ST11-KPC-2 strains were considered highly threatening clones. Infections caused by these prevalent strains were often difficult to treat, as they exhibited limited response to available treatment options (Zhang et al., 2017; Wang et al., 2018; Zhang et al., 2018). The SC35 strain, belonging to ST11, produced carbapenemase and possessed antibiotic resistance genes solely on the SC35 plasmid p1. Through sequencing analysis, it was discovered that the SC35 plasmid p1 carried the two repetitive tandem core structures for *bla*_KPC-2_ and *bla*_CTX-M-65_ in the MDR1 region, as well as *bla*_TEM-1_ with *rmtB* in the MDR2 region, and other resistance genes that overlapped with mobile genetic elements as In413 and Tn*As3* in the MDR3 region (**Figures 2**, **3**). The combination of resistance genes with various functions on both the chromosome and plasmids was what contributed to the challenging situation of having limited treatment options for the SC35 strain.

By comparing the SC35 plasmid p1 with closely related plasmids from Chengdu, China (pKPC2_130119, pKPC2_115069, pKPC2_020003, and pKPC2_095649), it was found that the SC35 plasmid p1 shared the highest similarity with pKPC2_130119 in the MDR1 and MDR2 regions. Both plasmids contained the two repetitive tandem core structures for *bla*_KPC-2_ and *bla*_CTX-M-65_, and *bla*_TEM-1_ with *rmtB* (forward or reversed). In contrast, the other plasmids only had one tandem core structure for *bla*_KPC-2_ and *bla*_CTX-M-65_, and *bla*_TEM-1_ with *rmtB* (forward or reversed). None of these plasmids, except the SC35 plasmid p1, had an MDR3 region (**Figure 2**). Therefore, The SC35 plasmid p1 showed a high level of similarity to the plasmid from Chengdu, China, with the only difference being the absence of an MDR3 region. Similarly, when comparing the SC35 plasmid p1 with other related plasmids (p21072329_1, pXHKP6–1, pBSI014-KPC2, and pDD01754–2) that had the *bla*_KPC-2_ core structure arranged separately from the *bla*_CTX-M-65_ core structure, it was observed that the *bla*_CTX-M-65_ core structure of SC35 plasmid p1 was more similar to that of pDD01754–2. On the other hand, the arrangement of the *bla*_KPC-2_ core structure in pDD01754–2 was identical to that of the SC35 plasmid p1 (**Figure 4A**). This finding was consistent with the previous discovery of F4_plasmid pA, which shared perfect identity with the SC35 plasmid p1 (Ma et al., 2023). Based on these observations, it was hypothesized that pDD01754–2, F4_plasmid pA, and SC35 plasmid p1 might be evolutionarily linked (**Figure 4B**).

## Conclusion

The presence of similarities in the structure of SC35 plasmid p1 with plasmids such as pKPC2_130119, pDD01754–2, and F4_plasmid pA suggests that these plasmids are interconnected in their evolutionary processes. The strain SC35, which carries multiple efflux pumps on its chromosome (such as *acrAB-tolC*) and harbors the two repetitive tandem core structures for *bla*_KPC-2_ and *bla*_CTX-M-65_, and *bla*_TEM-1_ with *rmtB* on a plasmid with overlapping structures of mobile genetic elements as In413, Tn*3*, and Tn*As3*, poses a significant threat. Effective preventive measures should be taken to stop the spread and prevalence of such antibiotic-resistant bacteria.

## Data availability statement

The datasets presented in this study can be found in online repositories. The names of the repository/repositories and accession number(s) can be found in the article/**Supplementary Material**.

## Ethics statement

This study was approved by the Ethics Committee of Taizhou Municipal Hospital, Zhejiang, China, and written informed consent was obtained from each of the participants in accordance with the Declaration of Helsinki. The rights of the research subjects were protected throughout, and we confirm that this study was conducted in our hospital. The use of human specimens and all related experimental protocols were approved by the Committee on Human Research of the indicated institutions, and the protocols were carried out in accordance with approved guidelines.

## Author contributions

LH: Data curation, Formal analysis, Funding acquisition, Investigation, Methodology, Project administration, Resources, Supervision, Validation, Visualization, Writing – review & editing. WW: Data curation, Formal analysis, Investigation, Methodology, Resources, Supervision, Validation, Visualization, Writing – review & editing, Software. LM: Data curation, Formal analysis, Investigation, Methodology, Resources, Software, Supervision, Validation, Visualization, Writing – review & editing, Project administration. DW: Data curation, Formal analysis, Investigation, Methodology, Project administration, Resources, Software, Supervision, Validation, Visualization, Writing – review & editing, Conceptualization, Funding acquisition, Writing – original draft. SL: Data curation, Investigation, Methodology, Resources, Supervision, Validation, Visualization, Writing – review & editing.

## Supplementary material

Supplementary Figure 1 Circos plots of SC35 plasmid p2, SC35 plasmid p3, and SC35 plasmid p4. The figure was established using *CGview v2.0.3* (https://github.com/paulstothard/cgview).

## References

[B1] Alcock B. P. Huynh W. Chalil R. Smith K. W. Raphenya A. R. Wlodarski M. A.. (2023). CARD 2023: expanded curation, support for machine learning, and resistome prediction at the Comprehensive Antibiotic Resistance Database. Nucleic Acids Res. 51, D690–D699. doi: 10.1093/nar/gkac920 36263822 PMC9825576

[B2] Boratyn G. M. Camacho C. Cooper P. S. Coulouris G. Fong A. Ma N.. (2013). BLAST; a more efficient report with usability improvements. Nucleic Acids Res. 41, W29–W33. doi: 10.1093/nar/gkt282 23609542 PMC3692093

[B3] Bortolaia V. Kaas R. S. Ruppe E. Roberts M. C. Schwarz S. Cattoir V.. (2020). ResFinder 4.0 for predictions of phenotypes from genotypes. J. Antimicrob. Chemother. 75, 3491–3500. doi: 10.1093/jac/dkaa345 32780112 PMC7662176

[B4] Boutet E. Lieberherr D. Tognolli M. Schneider M. Bansal P. Bridge A. J.. (2016). UniProtKB/Swiss-Prot, the manually annotated section of the UniProt KnowledgeBase: how to use the entry view. Methods Mol. Biol. 1374, 23–54. doi: 10.1007/978-1-4939-3167-5_2 26519399

[B5] Brettin T. Davis J. J. Disz T. Edwards R. A. Gerdes S. Olsen G. J.. (2015). RASTtk: a modular and extensible implementation of the RAST algorithm for building custom annotation pipelines and annotating batches of genomes. Sci. Rep. 5, 8365. doi: 10.1038/srep08365 25666585 PMC4322359

[B6] Cantón R. Coque T. M. (2006). The CTX-M beta-lactamase pandemic. Curr. Opin. Microbiol. 9, 466–475. doi: 10.1016/j.mib.2006.08.011 16942899

[B7] Clinical and Laboratory Standards Institute (CLSI) (2022). Performance Standards for Antimicrobial Susceptibility Testing. Thirty-two Informational Supplement. M100–S32. Wayne, PA: Clinical and Laboratory Standards. https://clsi.org

[B8] Edgar R. C. (2004). MUSCLE: multiple sequence alignment with high accuracy and high throughput. Nucleic Acids Res. 32, 1792–1797. doi: 10.1093/nar/gkh340 15034147 PMC390337

[B9] Feng Y. Zou S. Chen H. Yu Y. Ruan Z. (2021). *BacWGSTdb 2.0*: a one-stop repository for bacterial whole-genome sequence typing and source tracking. Nucleic Acids Res. 49, D644–DD650. doi: 10.1093/nar/gkaa821 33010178 PMC7778894

[B10] Frank J. A. Reichm C. I. Sharma S. Weisbaumm J. S. Wilson B. A. Olsen G. J. (2008). Critical evaluation of two primers commonly used for amplification of bacterial 16 S rRNA genes. Appl. Environ. Microbiol. 74, 2461–2470. doi: 10.1128/AEM.02272-07 18296538 PMC2293150

[B11] Hamidian M. Nigro S. J. (2019). Emergence, molecular mechanisms and global spread of carbapenem-resistant *Acinetobacter baumannii* . Microb. Genom. 5, e000306. doi: 10.1099/mgen.0.000306 31599224 PMC6861865

[B12] Li X. Z. Nikaido H. (2004). Efflux-mediated drug resistance in bacteria. Drugs 64, 159–204. doi: 10.2165/00003495-200464020-00004 14717618

[B13] Li X. Z. Plesiat P. Nikaido H. (2015). The challenge of efux-mediated antibiotic resistance in Gram-negative bacteria. Clin. Microbiol. Rev. 28, 337–418. doi: 10.1128/CMR.00117-14 25788514 PMC4402952

[B14] Li X. Quan J. Ke H. Wu W. Feng Y. Yu Y.. (2021). Emergence of a KPC variant conferring resistance to ceftazidime-avibactam in a widespread ST11 carbapenem-resistant *Klebsiella pneumoniae* clone in China. Front. Microbiol. 12. doi: 10.3389/fmicb.2021.724272 PMC8415713 34484166

[B15] Liu J. Xie J. Yang L. Chen D. Peters B. M. Xu Z.. (2018). Identification of the KPC plasmid pCY-KPC334: new insights on the evolution pathway of the epidemic plasmids harboring *fosA3*–*bla* _KPC-2_ genes. Int. J. Antimicrob. Agents. 52, 510–511. doi: 10.1016/j.ijantimicag.2018.04.013 29684435

[B16] Ma L. Wang W. Qu Y. Wang D. (2023). Characterization of the two repetitivetandem repeats for the KPC-2 core structures on a plasmid from hospital-derived *Klebsiella pneumoniae* . Sci. Rep. 13, 12049. doi: 10.1038/s41598-023-38647-z 37491538 PMC10368644

[B17] Moura A. Soares M. Pereira C. Leitão N. Henriques I. Correia A. (2009). INTEGRALL: a database and search engine for integrons, integrases and gene cassettes. Bioinformatics. 25, 1096–1098. doi: 10.1093/bioinformatics/btp105 19228805

[B18] O’Leary N. A. Wright M. W. Brister J. R. Ciufo S. Haddad D. McVeigh R.. (2016). Reference sequence (RefSeq) database at NCBI: current status, taxonomic expansion, and functional annotation. Nucleic Acids Res. 44, 733–745. doi: 10.1093/nar/gkv1189 PMC4702849 26553804

[B19] Pitout J. D. Nordmann P. Poirel L. (2015). Carbapenemase-producing *Klebsiella pneumoniae*, a key pathogen set for global nosocomial dominance. Antimicrob. Agents Chemother. 59, 5873–5884. doi: 10.1128/AAC.01019-15 26169401 PMC4576115

[B20] Qu Y. Wang W. Lu Q. Qiu J. Wang D. Ma L. (2023). Occurrence and characterization of plasmids carrying *tmexCD1-toprJ1*, *bla* _DHA-1_ and *bla_CTX-M-127_ *, in *Klebsiella pneumoniae* clincal strains. Front. Cell Infect. Microbiol. 13. doi: 10.3389/fcimb.2023.1260066 PMC10611489 37900313

[B21] Reygaert W. C. (2018). An overview of the antimicrobial resistance mechanisms of bacteria. AIMS Microbiol. 4, 482–501. doi: 10.3934/microbiol.2018.3.482 31294229 PMC6604941

[B22] Ruiz C. Levy S. B. (2010). Many chromosomal genes modulate MarA-mediated multidrug resistance in *Escherichia coli* . Antimicrob. Agents Chemother. 54, 2125–2134. doi: 10.1128/AAC.01420-09 20211899 PMC2863627

[B23] Shi D. Hao H. Wei Z. Yang D. Yin J. Li H.. (2022). Combined exposure to non-antibiotic pharmaceutics and antibiotics in the gut synergistically promote the development of multi-drug-resistance in *Escherichia coli* . Gut Microbes 14, 2018901. doi: 10.1080/19490976.2021.2018901 35014598 PMC8757474

[B24] Siguier P. Perochon J. Lestrade L. Mahillon J. Chandler M. (2006). ISfinder: the reference centre for bacterial insertion sequences. Nucleic Acids Res. 34, D32–D36. doi: 10.1093/nar/gkj014 16381877 PMC1347377

[B25] Stothard P. Grant J. R. Van Domselaar G. (2019). Visualizing and comparing circular genomes using the CGView family of tools. Brief Bioinform. 20, 1576–1582. doi: 10.1093/bib/bbx081 28968859 PMC6781573

[B26] Tansirichaiya S. Rahman M. A. Roberts A. P. (2019). The transposon registry. Mob. DNA. 10, 40. doi: 10.1186/s13100-019-0182-3 31624505 PMC6785933

[B27] Villa L. García-Fernández A. Fortini D. Carattoli A. (2010). Replicon sequence typing of IncF plasmids carrying virulence and resistance determinants. J. Antimicrob. Chemother. 65, 2518–2529. doi: 10.1093/jac/dkq347 20935300

[B28] Wang Y. He J. Sun L. Jiang Y. Hu L. Leptihn S.. (2023). IS26 mediated *bla* _CTX-M-65_ amplification in *Escherichia coli* increase the antibiotic resistance to cephalosporin *in vivo* . J. Glob. Antimicrob. Resist. 35, 202–209. doi: 10.1016/j.jgar.2023.09.018 37802302

[B29] Wang Q. Wang X. Wang J. Ouyang P. Jin C. Wang R.. (2018). Phenotypic and genotypic characterization of carbapenem-resistant Enterobacteriaceae: data from a longitudinal large-scale CRE study in China, (2012–2016). Clin. Infect. Dis. 67, S196–S205. doi: 10.1093/cid/ciy660 30423057

[B30] World Health Organization (WHO) (2014). Antimicrobial resistance: global report on surveillance (Geneva, Switzerland: WHO).

[B31] Xiang D. R. Li J. J. Sheng Z. K. Yu H. Y. Deng M. Bi S.. (2016). Complete sequence of a novel IncR-F33:A-:B-plasmid, pKP1034, harboring *fosA3*, *bla* _KPC-2_, *bla* _CTX-M-65_, *bla* _SHV-12,_ and *rmtB* from an epidemic *Klebsiella pneumoniae* sequence type 11 strain in China. Antimicrob. Agents Chemother. 60, 1343–1348. doi: 10.1128/AAC.01488-15 PMC4775989 26666939

[B32] Yamasaki S. Zwama M. Yoneda T. Hayashi-Nishino M. Nishino K. (2023). Drug resistance and physiological roles of RND multidrug efflux pumps in *Salmonella enterica*, *Escherichia coli* and. Pseudomonas aeruginosa. Microbiol. (Reading). 169, 1322. doi: 10.1099/mic.0.001322 PMC10333786 37319001

[B33] Yu K. Huang Z. Xiao Y. Gao H. Bai X. Wang D. (2024). Global spread characteristics of CTX-M-type extended-spectrum β-lactamases: A genomic epidemiology analysis. Drug Resist. Updat. 73, 101036. doi: 10.1016/j.drup.2023.101036 38183874

[B34] Zhai Y. Li D. Du P. Zhang Z. He Z. Guo Y.. (2021). Complete sequences of two new KPC-harbouring plasmids in *Klebsiella pneumoniae* ST11 strains in China. J. Glob. Antimicrob. Resist. 24, 114–120. doi: 10.1016/j.jgar.2020.11.023 33321214

[B35] Zhang R. Liu L. Zhou H. Chan E. W. Li J. Fang Y.. (2017). Nationwide surveillance of clinical carbapenem-resistant Enterobacteriaceae (CRE) strains in China. EBio Med. 19, 98–106. doi: 10.1016/j.ebiom.2017.04.032 PMC5440625 28479289

[B36] Zhang Y. Wang Q. Yin Y. Chen H. Jin L. Gu B.. (2018). Epidemiology of carbapenem- resistant enterobacteriaceae infections: report from the China CRE network. Antimicrob. Agents Chemother. 62, e01882–e01817. doi: 10.1128/AAC.01882-17 29203488 PMC5786810

