# Supplementary figures and images for: Emergence of a clinical Klebsiella pneumoniae harboring an acrAB-tolC in chromosome and carrying the two repetitive tandem core structures for bla KPC-2 and bla CTX-M-65 in a plasmid
Source: Front Cell Infect Microbiol. 2024 Jul 2;14:1410921. doi: 10.3389/fcimb.2024.1410921 (PMC11250256; doi:10.3389/fcimb.2024.1410921)

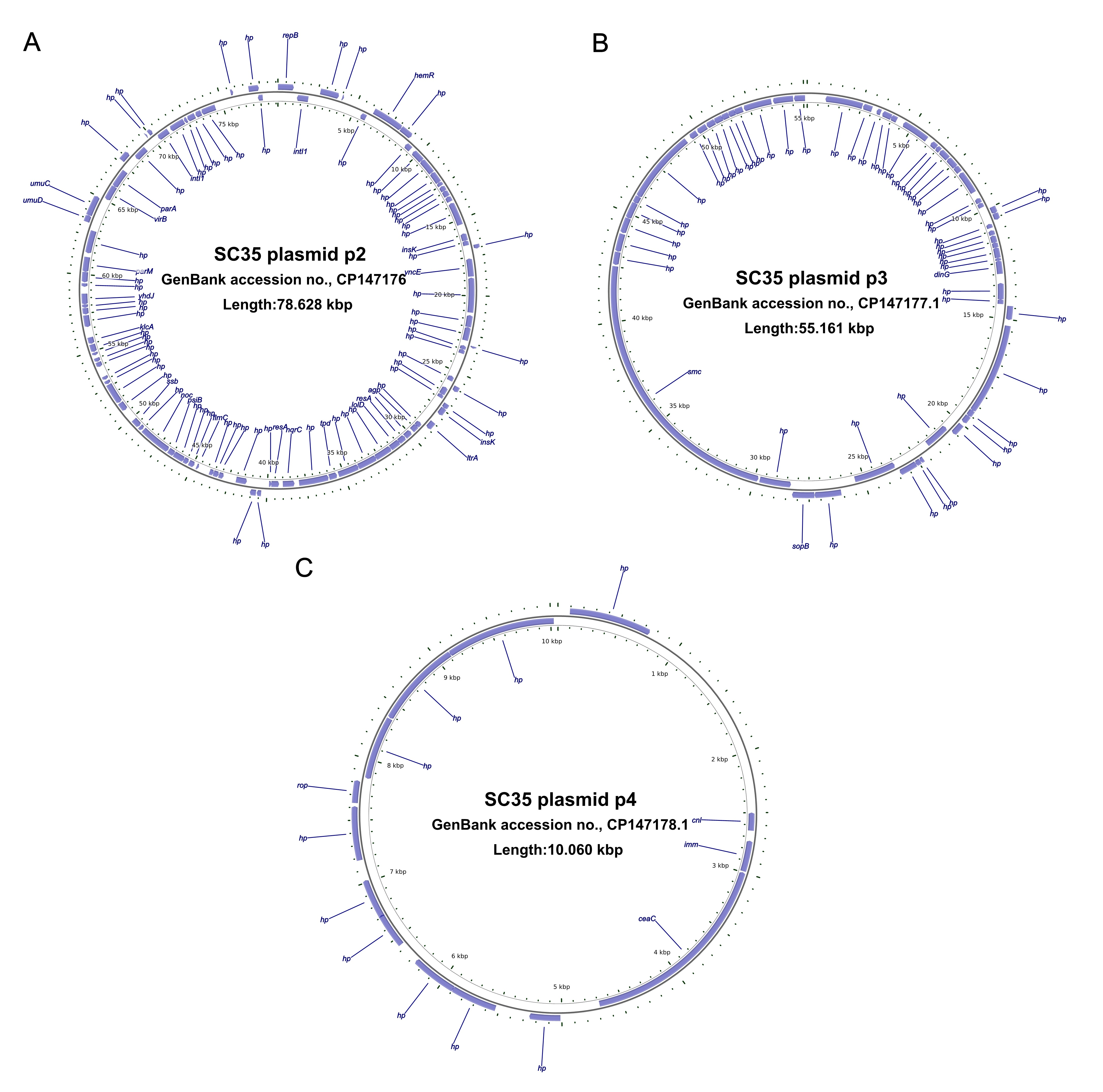

Supplement: Supplementary Figure 1 — Circos plots of SC35 plasmid p2, SC35 plasmid p3, and SC35 plasmid p4. The figure was established using CGview v2.0.3 (https://github.com/paulstothard/cgview). [file Image_1.tif]
